# Adhesion of the Placenta

**Published:** 1881-05

**Authors:** A. Cummings Air

**Affiliations:** L. R. C. P., London


### ADHESION OF THE PLACENTA.

BY A. CUMMINGS AIR, L. R. C. P., LONDON.

MORBID adhesion of the placenta to the uterine wall is fortunately of very unfrequent occurrence, but inasmuch as when it does happen it constitutes one of the most dangerous complications of labor, both from the great probability of its causing profuse post-partum hæmorrhage, and also from the risk of subsequent inflammation of the wound, the accurate diagnosis of this condition is of great importance, but according to the generally received teaching of modern text books, it is very difficult, if not absolutely impossible. Thus Dr. Barnes in his lectures on obstetric operations says: "You may suspect morbid adhesion if there have been unusual difficulty in removing the placenta in previous labors; if during the third stage the uterus contracts firmly, each contraction being accompanied by blood, and yet on following up the cord you feel the placenta still in utero; if on pulling on the cord, two fingers being pressed into the placenta at the root, you feel the placenta and uterus descend in one mass, a sense of dragging pain being elicited; if during a pain the uterine tumor do not present a globular form, but be more prominent than usual at the place of placental attachment." Dr. Playfair says: "The cause of adhesion is often obscure, but it most probably results from a morbid state of the decidua, which is produced by antecedent disease of the uterine mucous membrane; then the adhesion is apt to recur in subsequent pregnancies. * * There are no very reliable signs to indicate morbid adhesion of the placenta previous to the introduction of the hand." And Dr. Churchill: "The diagnosis is in almost all cases impossible until the extraction is attempted; a strong suspicion will be excited, however, by the occurrence of uterine contraction without extrusion of the after-birth. The previous history of the patient may in some degree confirm these suspicions; if she have suffered much pain in some fixed part of the uterus during pregnancy, it may have resulted from inflammatory action. Whenever we see a patient suffering thus, we should always ascertain by the stethoscope whether it is in the situation of the after-birth, so that we may be prepared for the consequences at the time of labor."

I have met with several cases of morbidly adherent placenta, during the last fourteen years, and am inclined to believe that the diagnostic problem be solved with almost absolute certainty; although from my experience being limited to so short a time, I would desire to write with all becoming modesty.

The diagnosis is, I think, to be founded upon two symptoms; one of which is mentioned by Dr. Churchill, the other by Dr. Barnes—viz., that at some period of pregnancy, generally between the third and fifth month, a fixed pain, generally of a dull aching character, is felt over some part of the uterus; and this is converted into a severe *dragging* pain when the patient attempts to turn over to lie on the side opposite to the placental side; so much so that patients, with an adherent placenta, will never (as far as my experience goes,) voluntarily lie on that side. This pain, I believe, to be of the same nature as that mentioned by Dr. Barnes, as being experienced when the cord is drawn upon; and is due to the dragging on the cord by the child, when from gravitation it sinks through the liquor amnii.

Theoretically, it may be objected to this explanation that usually the cord is sufficiently long to prevent any such dragging; but I think it will generally be found that when the cord is long, it is twisted around the neck or limbs of the child, and produces the same effect as a short cord would.

No history of this dragging pain on the patient's turning to the side opposite to the placental insertion will be obtained, when the retention of the after-birth is merely due either to the inertia of a wearied uterus, or from irregular contraction; if there is hæmorrhage in either of these cases, one would be justified in trying the effect of cold, compression, etc., before introducing the hand, but in cases of true placental adhesion, trying these and similar means leads to dangerous loss of precious time.—*London Lancet.*

